# Emergent inequality and self-organized social classes in a network of power and frustration

**DOI:** 10.1371/journal.pone.0171832

**Published:** 2017-02-17

**Authors:** Benoit Mahault, Avadh Saxena, Cristiano Nisoli

**Affiliations:** 1 Service de Physique de l’Etat Condensé, CNRS UMR 3680, CEA-Saclay, 91191 Gif-sur-Yvette, France; 2 Theoretical Division and Center for Nonlinear Studies, Los Alamos National Laboratory, Los Alamos, NM 87545, United States of America; 3 Institute for Materials Science, Los Alamos National Laboratory, Los Alamos, NM 87545, United States of America; Universidad Veracruzana, MEXICO

## Abstract

We propose a simple agent-based model on a network to conceptualize the allocation of limited wealth among more abundant expectations at the interplay of power, frustration, and initiative. Concepts imported from the statistical physics of frustrated systems in and out of equilibrium allow us to compare subjective measures of frustration and satisfaction to collective measures of fairness in wealth distribution, such as the Lorenz curve and the Gini index. We find that a completely libertarian, law-of-the-jungle setting, where every agent can acquire wealth from or lose wealth to anybody else invariably leads to a complete polarization of the distribution of wealth vs. opportunity. This picture is however dramatically ameliorated when hard constraints are imposed over agents in the form of a limiting network of transactions. There, an out of equilibrium dynamics of the networks, based on a competition between power and frustration in the decision-making of agents, leads to network coevolution. The ratio of power and frustration controls different dynamical regimes separated by kinetic transitions and characterized by drastically different values of equality. It also leads, for proper values of social initiative, to the emergence of three self-organized social classes, lower, middle, and upper class. Their dynamics, which appears mostly controlled by the middle class, drives a cyclical regime of dramatic social changes.

## Introduction

Ever since Vilfredo Pareto provided the earliest quantitative analyses of wealth distribution [1, 2], the inequality of societies has been a matter of debate and concern for economists, social scientists, and politicians alike. Its correlation, anti-correlation, or lack thereof, with growth is historically among the most debated issues in political economics [3–6]. Wealth inequality has recently caused growing concerns for the functioning of democratic institutions [7–9] and occupied the political debate [10].

While convincing econometric studies have reported increasing polarization of *income* in western societies [11, 12], more recent data document a worldwide increase in *wealth* inequality [12–16]. In general “wealth is unequally distributed, more so than wages or incomes” [12] and “Gini coefficients for wealth typically lie in the range of about 0.6–0.8. In contrast, most Gini coefficients for disposable income fall in the range 0.3–0.5” [17]. The Gini index [18] for the wealth of the entire world (and indeed of the USA) is estimated at a dramatic 0.8 with the bottom 50% of the world population owning 3.7% of wealth (ref. [17] and references therein).

It has been generally understood that wealth inequality follows income inequality, the delay corresponding to accumulation of savings among the upper-income strata [1, 19]. This process can be long (ref. [15] and references therein) impinging on issues of inherited wealth and social stratification [20]. However, faster, more direct pathways to wealth polarization might be possible in the fluid contemporary world where increasingly deregulated and sophisticated financial tools can provide new means for wealth transfer [21].

Simple examples abound. Most of the lower class’ wealth is stored in real estate, the acquisition of which involves financial instruments that effectively deflect towards other agents more than half of the painfully saved wealth, or indeed all of it, when epidemic foreclosures followed a bubble inflated by financial deregulation [22, 23]. Also, in the aftermath of a market crisis the least wealthy are at loss and cannot capitalize on the new low prices of assets for the ensuing market rebound as the wealthy can, providing a ratchet mechanism toward wealth inequality at each significant market oscillation. And indeed wealth inequality in the USA worsened after the 2007 financial crisis even though income inequality mildly ameliorated [24]. Finally, as deregulated financial instruments offset wage stagnation and the widening gap between productivity and living standards [12], a steady increase in household debt to finance consumption further contributes to wealth polarization [12, 25]. Indeed, the USA, at the forefront of financial deregulation, also tops most countries in wealth inequality, with a Gini index of ∼0.8 (worse than any African country except Namibia, as of year 2000) [17]. Furthermore, the American trend inversion concerning the share of the top 1% [13] began in the late 70s, closely tracking the deregulation of the financial market [26].

## Goals and structure of the paper

While the issue is certainly very much complex, it motivates a conceptualization of direct pathways to wealth accumulation/polarization, in a framework reflecting the essential features: power, inequality, frustration, and initiative. We seek here not (or not yet!) to provide precise quantitative predictions but rather to conceptualize these very complex issues within a simple model which nonetheless provides a rich phenomenology, one that lends itself to reasonable interpretations, analogous to those of social realities. Our aim here is thus to explore a new framework which can be later developed into a tool with predictive power.

In this work we thus explore how wealth inequality appears naturally in a minimal model of agents endowed with opportunities to grab or lose fractions of limited available wealth. The approach is inspired by our research in physics, chemistry and statistical mechanics [27–32] which provides rigorous means to predict statistical equilibria, in contrast to general equilibria in economics, and to also follow frustrated, out of equilibrium dynamics, which is relevant for inequality.

In particular, our framework allows us to compare subjective (frustration, satisfaction) as well as global (Lorenz curve, Gini index) measures of fairness, and study their interplay in the evolution of society.

Equipped with the aforementioned concepts we employ our framework to explore, at least qualitatively, the following rather relevant questions:

1. How is wealth distributed among the population in our model society?
2. How is frustration distributed among the population in our model society?
3. How are those distributions determined by the topology of the social network?
4. Does the distribution of frustration generally track the distribution of wealth? In other words, can we think of societies that are “unfair” in terms of wealth distribution, but at the same time expose a fair distribution of personal satisfaction?
5. How does the interplay of power and frustration affect—or indeed possibly drive—the social dynamics when agents are allowed to act upon their own frustration?

To explore these questions we consider three cases, which will be studied in detail in the section “Results”. We call the first the “Law of the Jungle” (of the homonymous subsection) as every agent can gain or lose wealth to every other, without constraints: there are no limits to the exchange. In such setting we look for the distribution of wealth which maximizes power in the society. This turns out to correspond, as one would expect, to the most savage inequality, with a lower class—made up of half or more of the population—comprised of utterly dispossessed individuals, with no wealth at all, and an upper class that sees all of its opportunities satisfied. This is true regardless of the particular distribution of opportunities in the society. It also follows that a seemingly reasonable recipe often touted by many, the one which typically reads “let’s increase opportunities for all”, does not really change anything in a “Law of the Jungle” setting: more than half of the society would still remain dispossessed.

In the second case (subsection: “Constrained, Static Societies”) we study how the imposition of constraints on the transfer of wealth can greatly ameliorate the inequality profile. We assume that each agent can only “play” within a network where it can exchange wealth with neighbors. We consider two classical cases (random network and scale free network) and show that they lead to rather different equality profiles, both in terms of wealth distribution and of personal satisfaction. Counterintuitively, the case least fair from the point of view of wealth distribution is also the fairest from the point of view of individual satisfaction.

The previous two cases pertain to static societies that equilibrate at maximum power. We have called “agents” the members of such society, but that was in a sense a misnomer in a framework where we were seeking the equilibrated state that optimizes power. Individual frustration/satisfaction played no role in determining the allocation of wealth.

In the third case, however, agents are in fact left free to “act”, in a kinetic model (subsection: “Emergent Social Classes and Kinetic Transitions in Market Coevolution”), thus establishing new connections with new neighbors, and severing old ones. A new global parameter is introduced, initiative, which measures the agents’ likelihood of acting on their frustration. We find that initiative controls the interplay of power and frustration. Above a critical value of initiative, society becomes dynamical, rather than converging to a more or less ameliorated inequality, with formation (and competition) of two social classes, and an alternation of times of equality and times of inequality. Equality can emerge, at least periodically, from the individual behavior of sufficiently motivated, frustrated agents.

## Methods

### Fundamental concepts

Given the multidisciplinary aim of this work, it might be useful to start by introducing some fundamental concepts to which we will return later in the manuscript. We begin with frustration.

Frustration is understood in everyday life as the emotional outcome of failure to achieve predetermined goals, despite proper investment in time and effort—not to mention one’s best intentions. Outside of the realm of emotions, it has come to signify situations in which full success is impossible. The failure—or indeed more commonly the only partial success—that originates frustration is often the result of limitations and constraints which are intrinsic and cannot be lifted—hence the bitter feeling.

Precisely for that reason, in Mathematics, where frustration can be more precisely characterized, it is understood as the result of a set of constraints which cannot be satisfied simultaneously. In Physics these constraints are generally related to the optimization of some energy, usually a pairwise interaction [28]. Thus, for instance, in the antiferromagnetic Ising model on a triangular lattice, it is impossible to find a configuration in which all the interactions are optimized, and thus all the neighboring spins are antiparallel to each other: an extensive number of couples of neighbors will have to be parallel (compromise) and they can be chosen with extensive freedom, resulting in a quite complex manifold.

Indeed in life too, frustration begets compromise, i.e. acceptance of partiality of success in the achievement of a goal. In life as in Physics, the set of multiple compromises among the numerous degrees of freedom of a problem typically composes into a lively manifold of configurations which exhibit a non-trivial collective dynamics. There, each change or movement has repercussions on the choice of compromise of every other agent. Instead, in absence of frustration, by definition everything converges to the optimal, and there is no dynamics, but only crystallized order.

In social sciences, where unlike in Physics, frustration cannot be easily quantified, nor indeed defined, it is typically expelled from modeling. Yet, few would doubt its role on social dynamics. In our model, inspired by Physics, we attempt to bring back frustration to the social realm from which the concept initially originated.

**Fig 1:**
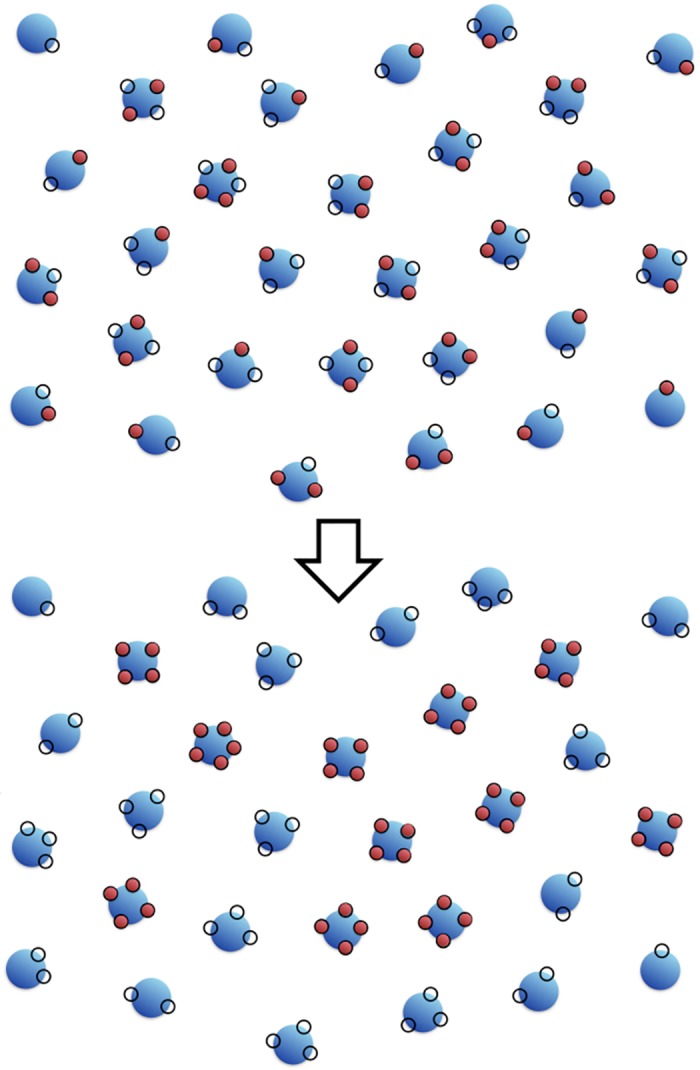
Schematic of the model. The “Law of the jungle” where every agent (in blue) can grab wealth (in red) from any other to fill its available opportunities (circles). The market power is optimized by a polarized society where agents with most opportunities get all their opportunities satisfied.

We consider a system of agents endowed with opportunities. Each agent has *z* opportunities to obtain units of wealth, where *z* changes among agents, and is distributed via *P*_*z*_, the fraction of agents endowed with *z* opportunities (see Fig 1). Each opportunity can be fulfilled by a unit of wealth, and the agent with *z* opportunities will possess *w* ≤ *z* units of wealth. Of course, what are objectively “opportunities” can also be seen, subjectively by the agent, as its “expectations”.

To frustrate the system we declare that there are twice as many expectations as there is wealth ($\bar{w}=\bar{z}/2$ where $\bar{w}$ and $\bar{z}$ are the average wealth and opportunities). Thus half of all the expectations go frustrated. Frustration is therefore built into the system and cannot be eliminated. Using this concept we can thus quantify the *individual satisfaction s* of an agent, defined as its gathered wealth *w* relative to its expectations *z*, and similarly its *individual frustration f* as the fraction of expectations that go unfulfilled, or

s=w/z (1)

f=1-s. (2)

Thus *f* = 1, *s* = 0 means complete frustration (no satisfied expectations), *f* = 0, *s* = 1 complete satisfaction (all expectations are satisfied). (Strictly speaking, it is redundant to use both these terms, as they are trivially related to each other. But, depending on the situation, one or the other seems much more natural.) While the system will be overall frustrated, a class of agents might very well be non-frutrated and thus quite satisfied, whereas another class might be extremely frustrated.

An obvious ingredient is still missing: the agent’s *power to aquire wealth*. We seek to model the cumulative advantage idea that power to acquire wealth itself derives from accumulated wealth (the so called “Matthew effect” [33, 34]). It follows that power must scale higher than linearly with wealth. We define the power P of an agent of wealth *w* as

P=w(w-1)/2. (3)

The physically inclined reader will recognize in the previous formula a total “energy” from the pairwise attraction of *w* units of wealth, rather than a power, although we call it “power” in reference to the layman understanding of “economic power”.

### Characterizations

In general we will study 〈*w*_*z*_〉, the distribution of average wealth for agents with *z* opportuinities, as well as its fluctuations within agents of the same level of opportunities/expectations, or $\sqrt{\langle w_{z}^{2}\rangle }$. From it we can compute the social motivation *m*_*z*_ = (〈*w*_*z*+1_〉 − 〈*w*_*z*_〉)/〈*w*_*z*_〉, a collective, averaged quantity describing the marginal increase in wealth for an increase in opportunities.

From *P*_*z*_ and 〈*w*_*z*_〉 we can then compute the classical—although coarser—economic measure of wealth distribution, the Lorenz curve for wealth [35], which graphs the cumulative share of wealth vs. cumulative population. A point (*x*, *y*) on the curve tells us the fraction *y*% of wealth owned by the bottom *x*% of the population. From it we can compute another classical indicator of equality, the Gini index *G*_*w*_ [18] to assess the global fairness of a society. We remind the reader that the Gini index is the normalized difference of the area between the Lorenz curve and the curve of perfect equality *y* = *x*, such that *G*_*w*_ = 0 denotes perfect equality in wealth distribution, while *G*_*w*_ = 1 denotes perfect inequality.

We employ such characterization tools as they are rather intuitive and ubiquitous in social sciences. However, we also realize that they are *global* indicators, somehow “coarser” than our framework, insofar as they disregard all the underlying *local* information pertaining to the individual opportunities/expectations of agents. This other set of information is however twofold important. Firstly, it provides very useful information on the agent’s “return on opportunity”: the global distribution of wealth might be irrelevant to the satisfaction of the individual, insofar most of its expectations are satisfied. Secondly, as we will see, it is reasonable to consider the individual satisfaction/frustration as a motor of society, which leads to interesting and complex dynamics.

We can recover the information lost in the Lorenz curve and the Gini index for wealth and by introducing average *individual* measures of fairness to describe the return on opportunity, such as average frustration $\bar{f}=\sum_{z}\langle f_{z}\rangle P_{z}$ and average satisfaction $\bar{s}$. From that we can also draw the Lorenz curve *for personal satisfaction or frustration* in addition to those for wealth, as well as the corresponding Gini index *G*_*s*_. A little thought shows that a Gini index of zero for personal satisfaction *G*_*s*_ = 0 implies that all agents have the same satisfaction (and thus frustration) equal to 1/2. All the agents have half of their opportunities satisfied, a generalization of what physicists call the “ice rule” [27, 29–32, 36–38].

## Results and discussion

### The Law of the Jungle

Consider a population of agents all endowed with varying number of “opportunities” *z*. These are defined as available slots, which might or might not be filled by *w* units of wealth, as in Fig 1. *P*_*z*_ is the normalized distribution of expectations and represents the market. As stated above, what are, objectively, “opportunities” can also be seen, subjectively by the agent, as its “expectations” and there are twice as many opportunities as there is wealth ($\bar{w}=\bar{z}/2$ where $\bar{w}$ and $\bar{z}$ are the average wealth and opportunities).

The distribution of wealth which maximizes the total power of the market can be found easily by filling with wealth the agents of largest opportunities and continuing in decreasing order of opportunity, until all wealth is stored. The result is a completely polarized society in which agents with opportunity above a critical value *z*_*c*_ have zero frustration and all their opportunities satisfied, whereas the others have nothing as in Fig 1. In such setting there is clearly no discernible “middle class”.

The critical opportunity *z*_*c*_ is implicitly defined by

$$\sum_{z<z_{c}}zP_{z}=\frac{\bar{z}}{2},$$ (4)

which follows from $\bar{w}=\bar{z}/2$. Now, $P_{z}z/\bar{z}$ is the distribution of expectations, that is the total number of opportunities for all the agents of equal opportunities. Thus, Eq (4) implies that all the agents with opportunities below the median fall in the class of the haves-not. Clearly the fraction of the utterly dispossessed depends on the structure of the market, or *P*_*z*_. Yet, a little thought shows that the dispossessed are always more than 50% of the population (they are exactly 50% when all the agents have the same number of opportunities [29]: even then, half of the population gets nothing).

In particular, one must always make the assumption that the overall wealth is large, as it means that the unit of transaction must be small compared with the overall wealth of the society. Then, for large $\bar{w}$, we can safely take the continuum limit on *z*, and find that a scaling *P*_*z*_ → *kP*_*kz*_ does not change the overall fraction of haves-not, as the critical opportunity also scales in Eq (4). In other words, in a Law of the Jungle setting, where power dominates and each agent can gain or lose wealth to anybody else, the fraction of dispossessed is scale invariant in the opportunities, and recipes often touted by politicians and experts, such as “increasing opportunities for everybody” are not viable policies in the absence of structural changes in the societal topology.

**Fig 2:**
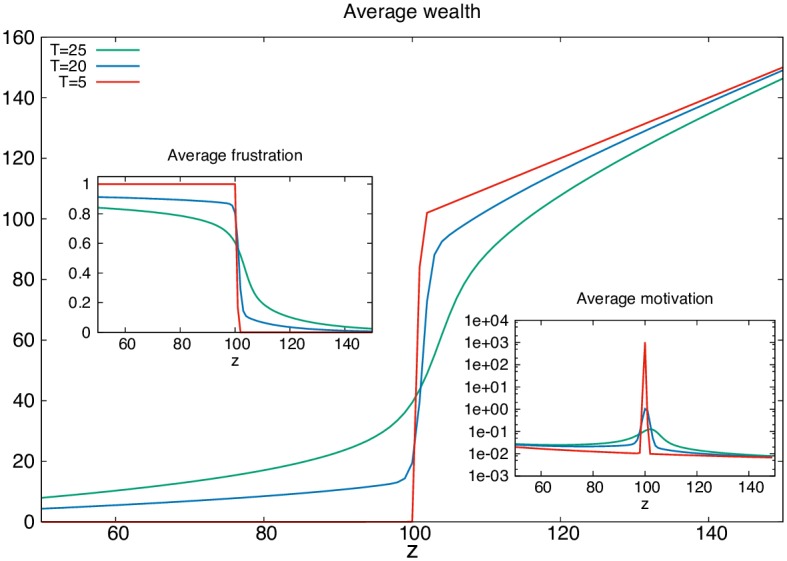
The distribution of average wealth 〈*w*_*z*_〉 vs. opportunities *z* at different temperatures in the Law of the Jungle setting of Fig 1 for a binomial distribution of opportunities of mean $\bar{z}=100$. The left and right insets plot respectively the average frustration and average motivation vs. opportunities *z*.

One way to grow a small middle class in this setting could be to make society less efficient in the optimization of power. We can introduce “thermal” disorder into the system via standard statistical physics techniques [29] already applied to social settings [39, 40], where a parameter *T*, an effective temperature, describes the deviation of the market from optimal behavior. Fig 2 shows that as expected, thermal disorder smears the curve, providing for a small middle class with opportunities centering around the critical value, situated among the haves and haves-not. Not surprisingly, the middle class is the only class endowed with motivation (Fig 2, right inset), as it is the only class which can experience a sensible marginal wealth increase following an increase in opportunity.

As society becomes more optimized for power (*T* → 0), the middle class shrinks to nothing, while its motivation skyrockets. (At very large thermal disorder power play no role and the ice-rule wins with 〈*f*_*z*_〉 = 1/2 leading to an egalitarian society, but that is a trivial and not very realistic case.)

We see that in this jungle setting a limited improvement in equality can only come from disorder that prevents complete optimization of power. However, we will see below, that even non-perfectly optimized jungles still lead to very strong polarization both in cumulative wealth and in individual frustration, at least compared to constrained societies.

### Constrained, static societies

We currently live in an age of financialized economy, where a vast portion of society’s wealth is deposited in financial markets and thus available for grabs by skilled agents. But until the mid 20^th^ century, even individuals of large opportunities could not grab wealth from just anybody else, as in the model described in the previous subsection. For instance the owner of a big company was mostly linked to his workers and could subtract from them part of the wealth they produced in the form of added value, yet was not similarly linked to many other economic agents.

**Fig 3:**
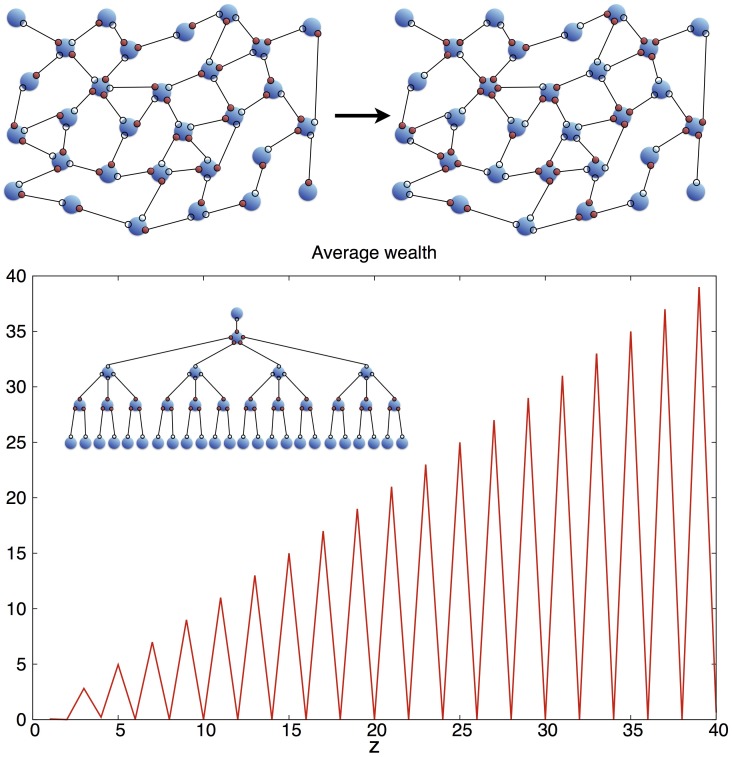
Top: same society as in Fig 1 but with hard constraints, which limit exchanges to pairwise action among agents and its optimized state. Bottom: The distribution of average wealth vs. opportunity optimizing power for a hierarchical fractal tree with *l* = 40 (The inset reports the case *l* = 5) as a (pathological) example of how hard constraints can severely frustrate wealth polarization.

It is thus natural to consider what effect the limitation of wealth transfer can have on wealth inequality. We consider pairwise transactions, by connecting agents as nodes of a network of shared edges. On each edge now sits the unit of wealth, and it can be grabbed or lost by the two nodes, as in Fig 3, top. The number of opportunities of an agent corresponds now to the coordination of the node (that is to the number of its connected neighbors) and *P*_*z*_ is therefore what in network theory is called the degree distribution of the graph. We will see that *P*_*z*_ together with *P*(*z*′|*z*), the conditional probability that an agent of opportunity *z* will have agents of opportunity *z*′ among its connected partners, might suffice to describe this model in most cases.

As network theory has been employed successfully in recent years to describe a number of complex systems, from the World Wide Web, to financial markets, neuronal connections, and the growth of cities [41–48], we apply it here to describe wealth transfer.

The following special, albeit possibly unrealistic, example can nonetheless demonstrate how a network setting can in principle radically modify the scenario of the previous subsection: consider the graph of Fig 3, a hierarchical tree where the agent with most opportunities *l* is connected to agents with opportunities *l* − 1 and so on. In the limit of large *l* we have a self-similar fractal and the distribution of wealth can be solved by iteration (see S1 Text). As Fig 3 shows, the relationship between wealth and opportunities for this network is completely different from the one obtained in the Law of the Jungle setting and reported in Fig 2. Indeed *it is not even monotonic*, precisely because each agent is connected to agents of similar opportunities. Therefore, as agents with high opportunities are linked to each other they cannot as a social class have all their opportunities satisfied.

While the previous, exactly solvable model serves as a useful counterexample, we now explore more realistic networks. We will seek for the state of maximum power, employing a standard Metropolis Monte Carlo algorithm. Each step of the algorithm corresponds to the transfer of a single unit of wealth among two randomly chosen connected agents, and is accepted if it increases the power, or else with probability exponentially decreasing in the decrease of power ($P^{\prime }-P$), proportional to $\exp[\left(P^{\prime }-P\right)/T]$. The degenerate manifold of maximum power is obtained when *T* → 0. We will confine ourselves to small values of *T* typically of order less than 1.

We will show the numerical results in the following section. Now we note that although the problem might seem analytically intractable, we find that the implicit mean field formula

$$\langle w_{z}\rangle =z\sum_{z^{\prime }}\frac{P(z^{\prime }|z)}{1+\exp\left[(\langle w_{z^{{}^{\prime }}}\rangle -\langle w_{z}\rangle )/T\right]}$$ (5)

excellently fits the numerical results (see S1 Text for a derivation) in the cases considered. Here *P*(*z*′|*z*), is the conditional probability that an agent of opportunity *z* has agents of opportunity *z*′ among its connected partners. Eq (5) can be understood in the following way: the average wealth of agents is proportional to their opportunity *z* times the fraction of connected partners of coordination *z*′, weighted with a mean thermal factor describing the probability of acquiring the unit of wealth.

For an optimized society (*T* → 0), and under the assumption that wealth increases monotonically with opportunities, Eq (5) returns the average satisfaction for an agent with *z* expectations as

$$\langle s_{z}\rangle =P\left(z|z\right)/2+\sum_{z^{\prime }<z}P\left(z^{\prime }|z\right).$$ (6)

Eq (6) tells us that the average satisfaction of a agent with *z* opportunities is given by the fraction of neighbors with equal or lower opportunities. We see that an agents has maximal satisfaction (*s* = 1) only when all its neighbors have fewer opportunities, which incidentally would lend credence to long held popular wisdom.

Eq (5), along with knowledge of *P*_*z*_ and *P*(*z*′|*z*), allows for the solution of the problem in the limit of a large society, which is often intractable numerically for a network.

### Random networks, scale free networks

We first test our framework on random graphs and scale free networks. Until about two decades ago, the former were assumed to be the most natural and common form of networks. The latter, however, have been more recently shown to successfully describe a wealth of structures, from the Internet [44], to alliance in industry [49] even to sexual encounters [50].

For random networks we employ the Erdős-Rényi model [51] where *N* is the number of vertices, *p* the probability of connection, and the degree distribution is binomial [$P_{z}=\left(\begin{array}{c} N-1\\ z \end{array}\right)p^{z}{\left(1-p\right)}^{N-1-z}$ and $P(z^{\prime }|z)=P_{z^{\prime }}$, see S1 Text for details]. The average opportunity is $\bar{z}=pN$ and in the thermodynamic limit of large *N* the degree distribution becomes Poisson if the average wealth $\bar{w}=\bar{z}/2=pN/2$ is kept constant. There, each agent is connected randomly to the others regardless of their coordination [$P(z^{\prime }|z)=P_{z^{\prime }}$]: the most coordinated agents are few and not much linked with other much coordinated agents (unlike the hierarchical tree described in the previous subsection). One would thus expect it to mimic the law-of-the-jungle case, with some added disorder.

We also build scale free networks via the Barabási-Albert preferential attachment algorithm [44] which has a scale free degree distribution *P*_*z*_ ∼ *z*^−3^ at large *z*, corresponding to highly correlated agents which are generally called hubs. Choosing the minimal coordination *m*, we then have $\bar{z}=2m$ (see S1 Text), and the average wealth is $\bar{w}=m$: unlike in the Erdős-Rényi model, all agents possess enough opportunities to own the average wealth. One would thus expect this network to be fairer. Interestingly, it is not.

**Fig 4:**
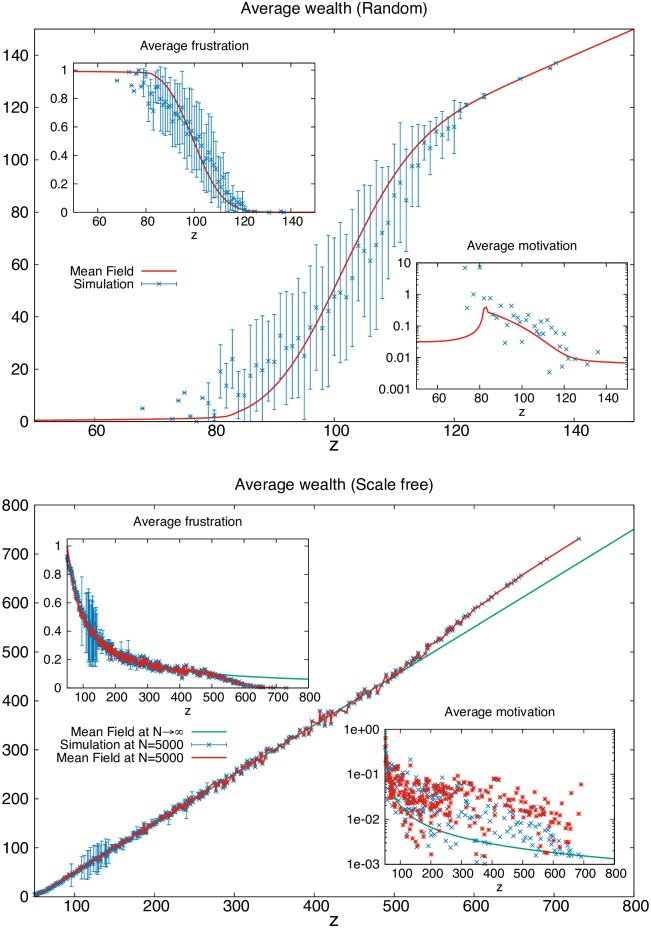
Results on random and scale free networks. Top: The distribution of average wealth 〈*w*_*z*_〉 vs. opportunities *z* for a random network with *N* = 1000 nodes and *p* = 0.1 probability of connection (thus average wealth $\bar{w}=50$) at *T* = 1, obtained via Metropolis simulation (in red the mean field formula). The left (right) inset represents the distribution of average frustration 〈*f*_*z*_〉 (motivation *m*_*z*_) vs. opportunities *z*. The bars denote fluctuations in wealth (and frustration) among agents endowed with the same opportunities due to the topological structure of the graph. Bottom: Same figures for a Barabàsi-Albert graph with minimum coordination $m=\bar{w}=50$ in a finite system (*N* = 5000 nodes) in the thermodynamic limit at *T* = 0.1. The green line describes the wealth allocation obtained by using *P*(*z*′|*z*) for the scale free network (reported in Methods) in the limit of a large society. Here, however, finite size effects cause a deviation of ∼ 5 × 10^−4^ agents among the richest. The red line is obtained via the mean field treatment using the *actual* conditional probability of the finite system, numerically generated, and reproduces the numerical results even better.

**Fig 5:**
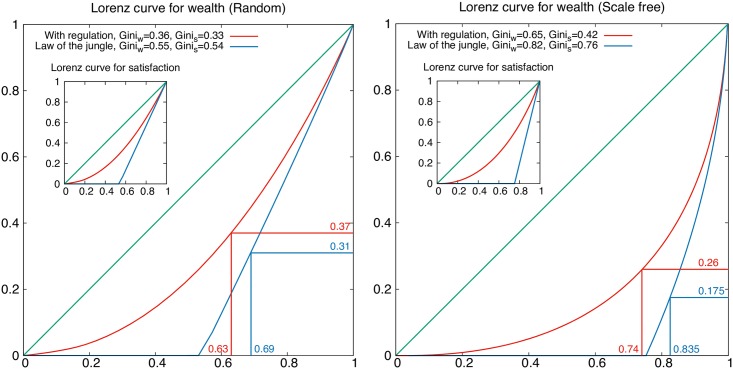
Results on random (left) and scale free networks (right). The Lorenz curves for cumulative wealth (in inset satisfaction) vs. cumulative population, their corresponding Gini coefficients, and Pareto squares with and without hard constraints, corresponding to the results shown in Fig 4.

Fig 4 reports both the results of Monte Carlo numerical simulations and of the mean field treatment described above, for these two networks. (We note incidentally that our mean field formula, Eq (5), excellently describes numerical results, and from Eq (5) it can be used to compute analytically Lorenz curves and Gini indices for large average wealth (Fig 5), in the limit of large average wealth $\bar{w}$.)

We see in Fig 4 that the allocation of wealth vs. opportunities/expectations is radically different in the two cases. In the random graph case one can distinguish three social classes, a lower class of very high frustration, a middle class of average opportunities and of frustration centered around 1/2, and an upper class of large opportunity and zero frustration, which is the least motivated. However due to the Poisson distribution of opportunities, most agents belong to the middle class and both the Lorenz curve and the low Gini index for wealth (*G*_*w*_ ≃ 0.36) and personal satisfaction (*G*_*s*_ ≃ 0.33) reflect this fairness, as shown in Fig 5. Furthermore, the middle class is characterized by the highest motivation and very large fluctuations of wealth for given opportunity (Fig 4, top, insets), something that Pareto already noted in real societies [1, 2]. Using our mean field formula we see that in the limit of a large society the average frustration is $\bar{f}=1/2$ (corresponding to the ice-rule on a network, as explained above) pointing to at least average fairness in frustration (see S1 Text).

Yet Fig 5 also shows that the same fair distribution of opportunities/expectations leads to a dramatically different scenario for what concerns wealth in a law-of-the-jungle case which has the same distribution of opportunities as that of the random network: while the Gini index is still decent (*G*_*w*_ ≃ *G*_*s*_ ≃ 0.5) and lower than the US Gini, in this case it proves to be a poor indicator of fairness: a look at the Lorenz curve shows that more than half of the population is completely dispossessed and unsatisfied.

The collective fairness of the Random network is inherited from its fairness in the distribution of opportunities, a narrower and symmetric distribution. As we will see in the next section, when the system is allowed to evolve out of equilibrium at the interplay of power and frustration, it will often try and sometimes succeed in recreating such a situation, typically by producing interdependent random networks.

In the scale free case, both numerics and our mean field treatment return, remarkably, a linear dependence of wealth as a function of opportunities and thus expectations, or $\langle w_{z}\rangle ≃z-\bar{w}$, as shown in Fig 4. Thus no clear class distinction can be deduced in Fig 4 and the average motivation simply increases as opportunities decrease as $m_{z}≃1/\left(z-\bar{w}\right)$.

Scale free networks are not known for being particularly egalitarian in their structure–indeed quite the opposite, they display preferential attachment to a few, largely connected hubs. Yet we see, remarkably, that at least the average wealth is accessible to everybody. Indeed everybody has relatively more opportunities than in a random network. Nonetheless Fig 5 reveals a worse Lorenz curve and Gini index (*G*_*w*_ ≃ 0.65) for wealth distribution among the population than in the random case. This can be understood from the hub-based topology of the random network. Few hubs are connected with agents of lower coordination, and they can take their wealth to generate more power.

And yet there is an interesting twist. While *collectively* less fair, the scale free network appears, in a more *subjective* way, reasonably fair. The Lorenz curve and Gini index for the individual satisfaction are not that much different from the random case: the society is more polarized in terms of plain wealth but not so much in the return on opportunities; the opportunities are simply distributed differently, with a few “hubs” getting most of them [44] and thus most of the wealth.

Here too a law-of-the-jungle setting returns a dramatic profile of inequality, indeed more dramatic than in the random case, with 75% of the population completely dispossessed and dissatisfied (Fig 5). By contrast, in the connected case, the fraction of the completely dispossessed goes to zero in the realistic limit of large average wealth $\bar{w}$. Indeed, it corresponds to agents of lowest opportunity $z=\bar{w}$, whose fraction is $2/(\bar{w}+2)$ [52] and goes to zero as $\bar{w}\to \infty$.

### Emergent social classes and kinetic transitions in society coevolution

Societies and markets are not static and seldom in equilibrium. While societies might have constraints for wealth transfer, these constraints are in general evolving. We can extend our framework by allowing agents to make decisions on the basis of their status to take, if not wealth, at least opportunities from others, or to break ties with neighbors and establish new relationships thus leading to an evolution–indeed a coevolution [53, 54]–of social topology. At the simplest level of modeling, our previous analysis on static markets suggests that such moves can be dictated by sheer power and also initiative to act on personal frustration.

As the total wealth is conserved, for any broken partnership a new one must emerge. In general the transition {*a* − *b*, *c*} → {*a* − *c*, *b*} involves three randomly grouped agents, *a*, *b*, *c*: *a* breaks ties with *b* and joins *c*, *b* loses an opportunity, *c* gains one, although this does not necessarily translate in gain or loss of actual wealth. Risk is involved.

**Fig 6:**
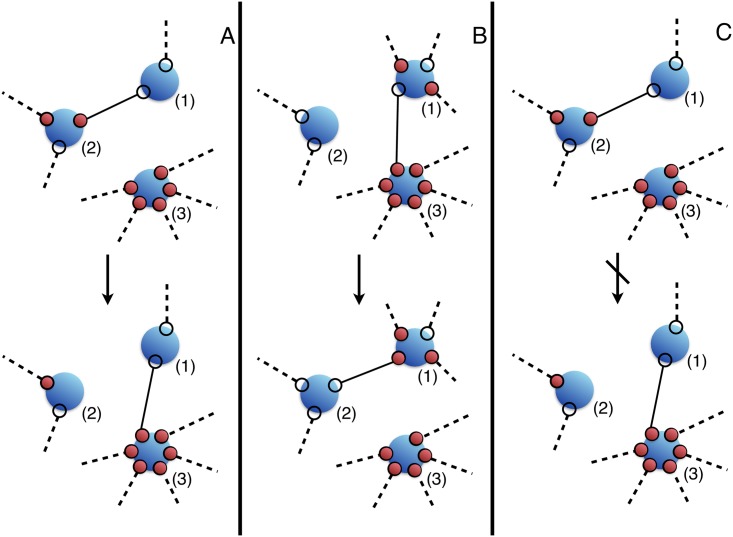
Dynamics of social interactions. Move A is promoted by power alone: (3) is much richer than (1) and (2) thus it will take the quantum of wealth they share. Move B and C on the contrary are promoted by frustration as in Eq (7). In B (3) is not frustrated but (1) and (2) are, thus the probability for them to reorganize their neighborhood is increased. Move C is suppressed by the social friction implicit in Eq (7).

**Fig 7:**
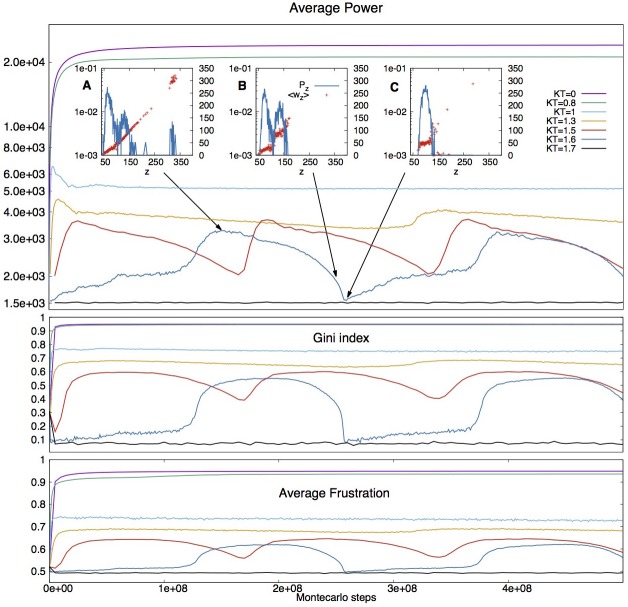
Effect of initiative on the coevolution of society. From top to bottom, average power, Gini index and average frustration at different values of initiative *K* evolving in time (Monte Carlo steps), starting from an equilibrated random graph with *N* = 1000 nodes and *p* = 0.1 attachment probability (thus average wealth $\bar{w}=50$, constant during the evolution), at minimal disorder *T* = 0.1. The A, B and C insets represent the average wealth (red, values on the right axis) and degree distributions (blue, values on the left axis) for *KT* = 1.6 at times 1.5 ⋅ 10^8^ (three social classes), 2.5 ⋅ 10^8^ (two classes) and 2.6 ⋅ 10^8^ (one class).

If the dynamics is based on power alone, then *c* will gain wealth as well as an opportunity if it is more powerful than *a*. This is depicted in transition A of Fig 6, which is promoted by a power-only dynamics where a move is accepted with a probability proportional to $\exp[\left(P^{\prime }-P\right)/T]$ where $P^{\prime }-P$ is the difference in power through the move, as before. Simulations show that at low disorder *T*, this process rapidly reaches an expected equilibrium, the best society for power: a Gini index close to 1 (Fig 7), complete inequality, and very few agents with extremely large opportunities, subtracting all the wealth from the remaining, low-coordinated network to which they are attached.

This suggest that if only power were involved in social evolution, not only the allocation of wealth of the society would converge to the results already presented in the Law of the Jungle model, but the very topology of the societal network would be reshaped for power optimization and thus concentration not only of wealth but also of opportunities.

However, it is reasonable to assume that in a more realistic setting each agent would be motivated to act on his/her frustration and also, if frustrated enough, to resist the action of the powerful. We can express such out-of-equilibrium effect by adding a new kind of probability for a move. We thus declare that the probability to accept a step {*a* − *b*, *c*} → {*a* − *c*, *b*}, where *a* is the active agent, *b* is the old partner, and *c* is the new partner, is proportional to the product of the previous, power-maximizing factor $\exp[\left(P^{\prime }-P\right)/T]$ times the extra factor

$$\exp\{K\left[\left(w_{b}-w_{c}\right)f_{a}+\left(w_{c}-w_{a}\right)f_{c}-w_{a}f_{b}\right]\},$$ (7)

where we call *K* “initiative” and all the wealths and frustrations of the agents are taken *in the initial configuration*.

As we can see, the initiative *K* gauges the effect of personal frustration on a move. Eq (7) is the product of three probabilities implying that a frustrated agent is more likely to change partner if the new partner is less wealthy than the old one. The more the new partner is frustrated, the more it is willing to acquire the new opportunity, but only if sharing wealth with a less wealthy partner. The old partner always resists losing an opportunity.

These three intentions, however, combine themselves to promote or suppress different transitions. For instance, in Fig 6 we see that transition B where two frustrated agents establish a new connection at the expense of a more powerful and less frustrated partner, is promoted. Agent (1) will be motivated to shift partnership away from agent (3) which is less frustrated and more powerful. Moreover, as (3) is much richer than (2) and un-frustrated, the greatly frustrated (2) will be motivated to accept the association with (1) even though the move proves not fortunate in terms of wealth. The factor in Eq (7) also suppresses transition C of Fig 6 because (1) and (2) are still frustrated and this move would have a negative effect on (1) who has fewer chances to get the unit of wealth at the end.

We note that the extra term due to frustration represents an out of equilibrium drive, as it is based on the initial configuration, rather than on the difference of configurations through the move: each move carries risk, and there is no assurance that a switch in partnership will bring wealth to the agent. (S1 Text provides a table of Eq (7) for extreme cases.)

We perform out of equilibrium Monte Carlo simulations via the algorithm described above. The initial state is a random network, however, using a scale free network does not change the picture (see S1, S2 and S3 Animations for the same kinetics applied to both networks). We believe this process is independent of the initial condition, after a sufficient amount of time.

**Fig 8:**
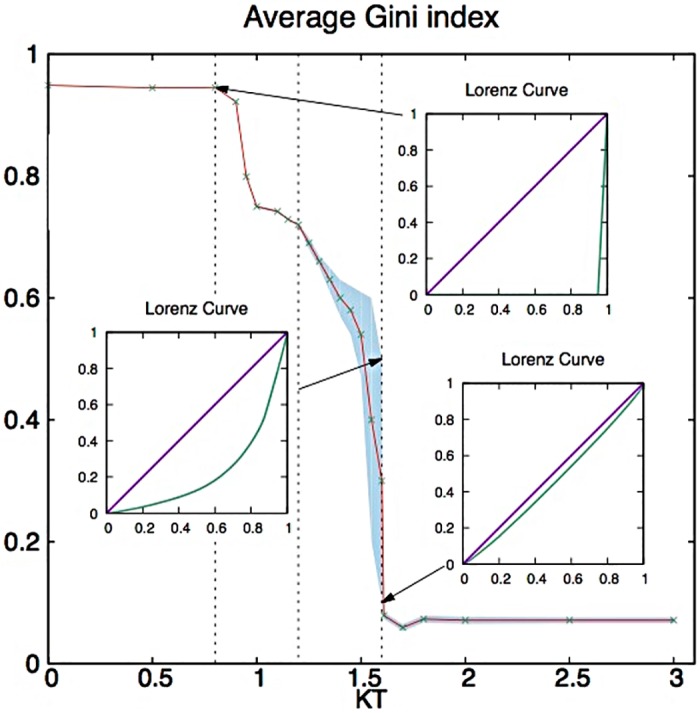
The mean Gini index as a function of initiative *K* (for *T* = 0.1), and its kinetic transitions (the blue shaded area corresponds to the periodic regime). The insets show the Lorenz curves at (*KT* = 0.8, *G* = 0.94), (*KT* = 1.6, *G* = 0.55) and (*KT* = 1.6, *G* = 0.09). The dashed lines distinguish four kinetic regimes: from left to right the power-dominated, the softened power, the periodic, and the egalitarian regimes.

**Fig 9:**
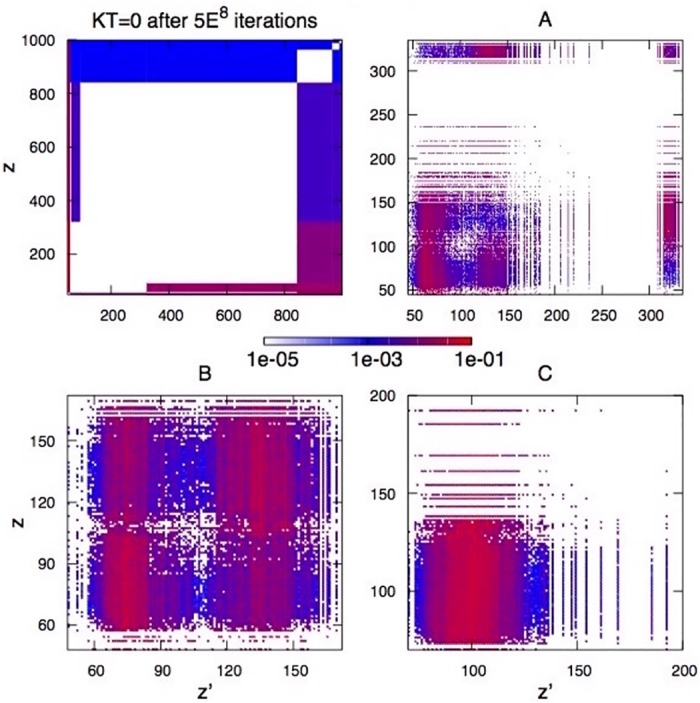
Density plots of the conditional degree distribution *P*(*z*′, *z*) of the graphs for insets A, B, C of Fig 7, and also in the *KT* = 0 case after 5 ⋅ 10^8^ iterations, showing that in the absolute-power regime all of the most coordinated agents are principally connected to the lower class.

Figs 7, 8 and 9, and the S1–S3 Animations show results for simulations at different values of initiative *K* at fixed *T* = 0.1. They reveal intriguing kinetic transitions. In Fig 7 at *K* < 8 we observe the same power-dominated regime described above, and the evolution rapidly converges to a topology optimized for power and characterized by extreme inequality. When 8 < *K* < 13 we have a regime of ameliorated inequality, where initiative and frustration can at least win points over power. The system still converges, but to a structure of lower power and lower Gini index for wealth (in the range 0.7–0.9, observed for instance in USA and UK). More importantly, in this regime, the higher is the initiative, the lower is the Gini index for wealth at convergence.

However, as initiative *K* increases above $K_{c_{1}}≃13$ we enter a cyclical behavior, which points to dramatic social changes. The network *does not converge* to a stable society anymore, but instead both power and the Gini index oscillate around stable values. Furthermore, those values decrease sharply as *K* (initiative) increases. In this regime the Gini index spans a range between a very low minimum value of ∼ 0.1 and a maximum of slightly more than 0.6 (in this range falls Japan with a Gini for wealth of 0.55). Interestingly, as *K* increases so does the amplitude of oscillations. That is because the minimum Gini decreases much faster than the maximum.

This window of oscillation abruptly collapses at large enough initiative, corresponding to $K_{c_{2}}≃16$. For *K* > 16 the market converges rapidly again, but this time to a regime of self-organized equality, which corresponds to a random network with a very small Gini index (∼ 0.1).

In Fig 8 we plot the Gini index of convergence (line) or the oscillating Gini index (blue shaded regon) as a function of initiative *K* at fixed *T* = 0.1, which reveals four kinetic phases. In increasing order of initiative *K* we have: a stable, power-dominated regime; a stable, power-ameliorated (by initiative) regime; then a regime of social changes (here periodic); and finally a stable, completely egalitarian regime.

For the cyclical regime of social changes, Figs 7 and 9 tell an interesting tale of three social classes. A maximum in power corresponds to *a self-organizazion into very distinct lower, middle and upper classes* with respect to opportunities and thus wealth. We remark that in no way this structure is built into the assumptions, it is instead emergent from the coevolution of the society.

From the insets of Fig 7, and from the conditional degree distribution in Fig 9, these classes appear as interdependent networks [43], each of them resembling a random network, possibly for maintained fairness *within* the social class. Looking at *P*(*z*′|*z*) in Fig 9 we notice that the upper class draws wealth from the middle class, which draws wealth from the lower class.

One possible interpretation of this social co-evolution could be the following. Motivated frustration works first through the middle class slowly bringing down the upper class, and thus improving equality among the top owners, which corresponds to change in shape of the Lorenz curve although with little improvement in the Gini index (Insets A and B in Figs 7 and 9, and S1–S3 Animations). As the middle class broadens, it finally merges with the remnants of an upper class whose wealth has been eroded (inset B, Fig 7). After that, quite suddenly, the two remaining classes coalesce realizing the more equal configuration of the cycle, a single random network of minimal Gini index. There, the value of the Gini index might be as low as *G*_*w*_ ≃ 0.1(inset C in Figs 7 and 9) in proximity of critical initiative $K_{c_{2}}$.

Yet the society is now so fair that nobody is particularly more frustrated than the others. Then, black swans of richness appear out of infrequent fluctuations. They are not connected between themselves but can draw wealth from the larger, equalized class (see inset C in Figs 7 and 9). The latter is now reasonably satisfied and deprived of sufficient frustration to oppose a power grab. The black swans of richness can thus win against the other agents, as the lack of frustration of the latter is insufficient to oppose the large power of the former. Thus, an upper class begins reforming again, although it is so tiny that the Gini index is left unchanged. In that situation, at the lower values of initiative *K*, a middle class appears almost immediately, the society returns to being suddenly polarized, and the cycle restarts.

The “time of equality” is not long lived at low initiative. But for initiative *K* close to the transition to an egalitarian regime ($K_{c_{2}}$ in Fig 8) the time of equality can stretch as long as the time of inequality, as an upper class forms very slowly (Fig 7) rather than suddenly; in both cases it appears that a middle class forms only after an upper class has reached a ‘critical mass’. Then as the middle class forms, it brings back the time of inequality typical of the 3-class system, as it continues its erosion of the upper class power, without much initial effect on the Gini index (see S1 Animation).

In our framework it appears that the birth of an upper class promotes a middle class, and thus indirectly pushes the lower class into poverty. However the middle class works to bring down the upper class. Indeed the lower class has frustration but no power. The upper class is powerful yet satiated. It is the middle class, with its mixture of both sufficient power and frustration, that on one hand impoverishes the lower class by taking its wealth (while at the same time being deprived by the upper class), on the other hand brings down the upper class, by taking its opportunities, in a process that improves equality only among the top owners, as it can be seen in the S1–S3 Animations.

**Fig 10:**
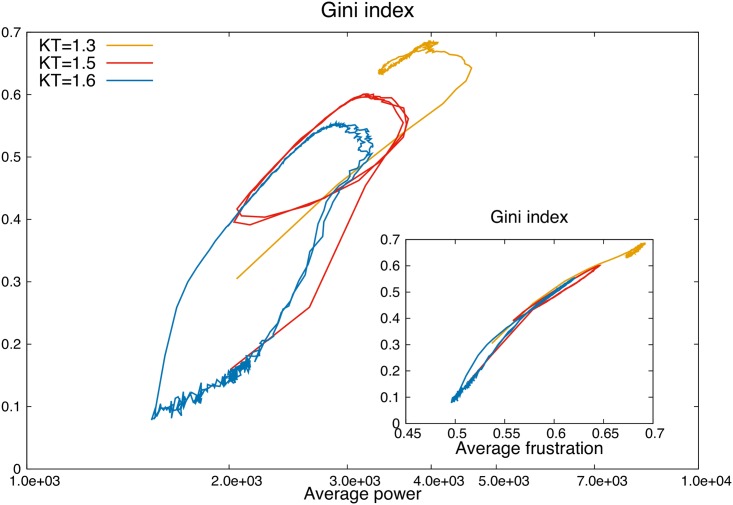
Hysteresis of Gini index vs. average power. Gini index vs. average power and frustration for different initial parameters in the oscillatory regime. We see a hysteresis cycle (counterclockwise) in which the Gini index lags behind power. The area of the hysteresis grows with the amplitude of the oscillations as we approach the transition to the frustration dominated regime. Inset: Gini index vs. average individual frustration shows almost no hysteresis, even though one is a collective measure of wealth distribution, the other a subjective measure of relative dissatisfaction.

This complex 3-body behavior is reflected in an increasingly (in initiative *K*) hysteretic behavior of the Gini index which lags behind power (see Fig 10). In the non-cyclical regimes, the Gini index is simply a monotonically increasing function of power. Instead in the cyclical regime the Gini index remains high as power decreases, and then low as power increases. The lag increases with higher social initiative *K*, as shown by the corresponding increase of the hysteretic area, which is zero at *K* = 13 and reaches a maximum at *K* = 16, in proximity of the transition to the egalitarian regime. The area of the hysteresis cycle is thus a good candidate to quantify social friction.

Interestingly the average frustration *tracks the Gini index almost without hysteresis* through the cycles (Fig 10, inset). This is not trivial. Indeed, while the frustration is a *subjective* measure of personal dissatisfaction and can perhaps be related to indices employed in analytic social psychology [55], the Gini index for wealth is a well defined, well known *collective* measure of wealth allocation: it is perhaps reasonable that these subjective and collective parameters should be in such direct correspondence in the dynamics that drives society’s evolution, but it is certainly not obvious.

## Conclusion

To conceptualize the more dynamical contemporary societies, we have presented a simple, heuristic, and qualitative model to gain insight into the problem of wealth allocation, alternative to the accepted frameworks based on generational social stratification. Within our model, complete deregulation on wealth transfer brings in savage inequality, efficiently dispossessing more than 50% of the population.

Static regulation of a society, expressed here through limitation on wealth transfer, considerably ameliorates the situation, even eradicating the completely dispossessed, and leading to remarkably fair Gini indices for personal satisfaction, even though the collective equality might differ.

Driven by the interplay of power and personal frustration/satisfaction, the coevolution of a society does not converge to just any Gini index value. Depending on individual initiative, it can return either of two extremes: more or less ameliorated inequality (Gini > 0.7) or almost complete equality (Gini = 0.1). In the middle lies a cyclical regime of oscillating equality, characterized by the emergence of three distinguishable classes, upper, middle and lower, whose mutual “3-body” interaction drives the cyclicity. There, periodically, during long times of relative inequality the middle class works patiently and relentlessly to bring down the upper class, and to merge with it. However, when a single egalitarian class forms for a brief time, it is soon disrupted by the appearance of the black swans of richness whose power, now competing against unfrustrated and thus demotivated agents of an egalitarian class, scores easy victories. A new time of inequality is brought in as a new middle class emerges with the rise of the upper class.

Interestingly, we see that equality can emerge from frustration through collective behavior, when individual initiative is high enough.

In our model, in societies with a Gini index larger than circa 0.9 individual initiative does not affect the wealth distributions. In societies with a Gini index between 0.9 and 0.7, initiative cannot achieve any dramatic global change and are thus rather stable societies, albeit quite unequal. There, personal initiative can only soften the effect of power and mildly ameliorate inequality.

More equalitarian societies, with Gini index less that 0.7, enter instead into a more turbulent dynamics in their collective co-evolution, where equality itself follows from a competition between power and initiative, motivated by frustration, which can drive major social changes.

More generally one might deduce that equality can be improved either by social engineering of a static and proper social topology or more realistically by dynamic, emergent reshaping of the society via sufficient individual initiative: this involves taking action on personal frustration, but also individual resistance to power-moves.

Even there, however, equality proves not stable, as the disappearance of frustration removes its fundamental promoter (except in the perhaps unrealistic regime where personal initiative is so high as to trump a resurgence of power concentration, leading to a completely egalitarian setting). Perhaps a key element in preventing the cyclical return of inequality in the three class system would be *memory*, which is absent from our framework. But is it present in society?

Finally, in future work we will explore individual trajectories of agents during society co-evolution, to conceptualize upward/downward class mobility. We will also extend the framework to allow for wealth creation, and discuss whether a gradient of wealth inequality impedes or promotes growth. There, a preferential attachment to the wealthy, which presumably hold more power to create new wealth in a partnership, can be introduced as a third degree of freedom, in addition to frustration and power, and might perhaps lead to the production of interdependent scale free networks.

## Supporting information

S1 Text Supporting information text. (PDF)

S1 AnimationIn this clip we illustrate the co-evolution of a graph for different initiative (*KT* = 0.8, 1, 1.6, 1.7) which corresponds to the four different observed regimes: Power dominated, power damped, cyclical and frustration dominated.We started the Monte Carlo simulations with a power-equilibrated random graph of 1000 nodes and average wealth $\bar{w}=50$. The left panel corresponds to the temporal evolution of the average power, the Gini index and the average frustration in the system. From left to right the different columns show the shape of the degree distribution, the distribution of wealth vs. opportunity with error bars corresponding to the fluctuations due to the topological structure of the network and the Lorenz curve.(GIF)

S2 Animation To assess the dependence of the co-evolution from initial condition we compare, in these two movies, the cases of *KT* = 1.5 starting from two different initial conditions. In this case we have an Erdős Rényi graph with 1000 nodes and average wealth 50, the second is a non-equilibrated Bàrabasi-Albert graph with the same parameters. On top left we show the degree distribution of the networks, the top left corresponds to the wealth vs. opportunity distribution, bottom left is the evolution of power with time and bottom right is the Lorenz curve with the Gini index. We see that both these initial conditions and those of S2 Animation lead to an oscillatory regime with in fact quite the same observable distribution. This and other simulations allow us to speculate that the dynamical evolution provided by our algorithm is not strongly dependent on the initial conditions.
(GIF)

S3 Animation To assess the dependence of the co-evolution from initial condition we compare, in these two movies, the cases of *KT* = 1.5 starting from two different initial conditions. In this case we have a non-equilibrated Bàrabasi-Albert graph with the same parameters. On top left we show the degree distribution of the networks, the top left corresponds to the wealth vs. opportunity distribution, bottom left is the evolution of power with time and bottom right is the Lorenz curve with the Gini index. We see that these initial conditions lead to an oscillatory regime with in fact quite the same observable distribution. This and other simulations make us speculate that the dynamical evolution provided by our algorithm is not strongly dependent on the initial conditions.
(GIF)

S1 Fig The Gini index and the proportion of the completely dispossessed in the population vs. the average wealth for the different cases we have studied. (TIFF)
