# Supplementary material for: Emergent inequality and self-organized social classes in a network of power and frustration
Source: PLoS One. 2017 Feb 17;12(2):e0171832. doi: 10.1371/journal.pone.0171832 (PMC5315399; doi:10.1371/journal.pone.0171832)
Supplement: S1 Text — (PDF) [file pone.0171832.s001.pdf]

Supporting Information

Contents

I. **Heuristic derivation of the mean field formula** 1

II. **Exact solution for the hierarchical fractal tree** 1

III. **Analytical study of equality for the random and scale free graphs** 2
- A. Random graph 2
- B. Scale free graph 2
  - 1. Connected graph 2
  - 2. Law of the jungle 3

IV. **Effect of frustration-based initiative on co-evolution.** 3

V. **Caption of Animations** 3
- A. Animation 1 3
- B. Animations 2 & 3 3

References 4

I. HEURISTIC DERIVATION OF THE MEAN FIELD FORMULA

Consider a set of agents whose opportunities are linked pairwise as in Fig 1. We can immediately write an equation for the wealth w_i of a node i whose neighborhood is noted ∂_i as

$$w_i = \sum_{j \in \partial_i} p_{ji}, \tag{1}$$

where p_{ji} is the probability for the wealth of the edge between i and j to belong to i and depends on T, w_i and w_j in a nearest neighbor approximation. At low temperature if the power associated to the vertex i is larger than the one related to j then $p_{ji} \to 1$, in the opposite case $p_{ji} \to 0$. If we isolate a link ij in the network and we fix its polarization we have a two-states system which is defined by: state $\mathbf{1} = (w_i + 1, w_j)$ and state $\mathbf{2} = (w_i, w_j + 1)$. We can write an equation of conservation for the probabilities α_1 and α_2 respectively associated to the states **1** and **2**

$$\alpha_1 + \alpha_2 = 1. \tag{2}$$

We also can express the ratio α_1/α_2 as a function of the powers of the states **1** and **2** using thermodynamic theory as

$$\frac{\alpha_1}{\alpha_2} = e^{(\mathcal{P}_1 - \mathcal{P}_2)/T} = e^{(w_i - w_j)/T}, \tag{3}$$

which with (2) leads to an integral expression for w_i

$$w_i = \sum_{j \in \partial_i} \frac{1}{1 + e^{(w_j - w_i)/T}}. \tag{4}$$

We are interested in the average wealth of a node of a certain degree z, which is given by

$$\langle w_z \rangle = \sum_{w=0}^{z} w \rho_w^z, \tag{5}$$

with ρ_w^z the probability for a node of degree z to have wealth w. We assume in a first approximation that this probability is given by $\delta_{w,\langle w_z \rangle}$. Moreover, as this formulation assumes that every agent of same coordination behaves in the same way it is relevant to suppose their neighborhood to be well defined by the conditional degree distribution of the network. Then we get an expression for the average wealth of an actor of a certain coordination z

$$\langle w_z \rangle = z \sum_{z'} P(z'|z) \frac{1}{1 + e^{(\langle w_{z'} \rangle - \langle w_z \rangle)/T}}. \tag{6}$$

Firstly, we remark that the formula works for a lattice of single coordination $\bar{z}$. In this case $P(z'|\bar{z}) = \delta_{\bar{z},z'}$, so we directly get that $\langle w_{\bar{z}} \rangle = \bar{z}/2$ and obtain the classic ice rule [1, 2]. Secondly, we notice that when $T \to \infty$, $\langle w_z \rangle \to z/2$, as one would expect in a society where exchanges are completely inefficient. Finally, Eq. (6) obeys wealth conservation ($\bar{w} = \bar{z}/2$). To see this it is convenient to define the topological charge of a node of degree z as $\langle q_z \rangle = 2\langle w_z \rangle - z$ such that $\bar{q} = \sum_z P_z \langle q_z \rangle = 0$. Equation (6) returns for $\langle q_z \rangle$

$$\langle q_z \rangle = \frac{z}{2} \sum_{z'} P(z'|z) \tanh\left(\frac{z - z' + \langle q_z \rangle - \langle q_{z'} \rangle}{2T} \right). \tag{7}$$

Then the conservation of total wealth ($\sum_z P_z \langle q_z \rangle = 0$) follows as the hyperbolic tangent is antisymmetric in z, z', while the quantity $z P_z P(z'|z)$, representing the total number of links between the nodes of coordinations z' and z, is symmetric.

Equation (6) characterizes the network as described only by $P(z'|z)$. Then the mean field solution of the problem is found by solving the nonlinear system of Eq. (7). Clearly in most of the cases it cannot be done analytically.

II. EXACT SOLUTION FOR THE HIERARCHICAL FRACTAL TREE

The tree described in the main text has two interesting properties: First, it supports coordinations extending to

infinity (in the thermodynamic limit $l \to \infty$), secondly more coordinated agents are fewer in number as to return finite integrals in the definition of the average coordination and other relevant observables. Finally, it serves in our framework as a strong counterpart to the unconstrained case: indeed for this lattice the constraints are so dramatic as to break down correlations between wealth and opportunities. While opportunities are still needed to amass wealth, it is perfectly possible to have very large opportunities and be very poor.

Let us consider a tree with l levels. The node at the top has coordination $l+2$, then it generates $l+1$ nodes of coordination $l+1$ which generate themselves l neighbors of coordination l and so on, until nodes of degree one are reached. Here the number of nodes of coordination z is equal to $(l+1)!/(z-1)!$ and so the total number of agents is equivalent, in the thermodynamic limit, to $\nu(l+1)!$ where $\nu = e-1$. Moreover, knowing that a node of degree z is connected one time with a node of degree $z+1$ and $z-1$ times with a node of degree $z-1$ we can compute P_z and $P(z'|z)$ for that tree:

$$P_z = \frac{1}{\nu(z-1)!}, \tag{8}$$

$$P(z'|z) = \frac{1}{z}\left[\delta_{z+1,z'} + (z-1)\delta_{z-1,z'}\right], \tag{9}$$

which are used to compute the average wealth with the mean field formula and to obtain the bottom right panel of Fig 1. The hierarchical tree, because of its fractal structure, can be solved exactly by iteration on its self-similarity. Let us call Z_l the partition function of such a tree with l levels. Then we define Z_l^+ and Z_l^- as the partition functions of the system with respectively the wealth shared by the agent on the top of the tree belonging to the one of degree 1 and $l+2$ ($Z_l = Z_l^+ + Z_l^-$). As a generation-l tree is simply formed with $l+1$ generation-$(l-1)$ trees and a coordination-1 agent we can write a recursive system of equations for Z_l^+ and Z_l^-

$$\begin{cases} Z_l^+ = \sum_{i=0}^{l+1} \binom{l+1}{i} p_i p_1 {Z_{l-1}^+}^{i} {Z_{l-1}^-}^{l+1-i}, \\ Z_l^- = \sum_{i=0}^{l+1} \binom{l+1}{i} p_{i+1} {Z_{l-1}^+}^{i} {Z_{l-1}^-}^{l+1-i}, \end{cases} \tag{10}$$

where $p_i = \exp[i(i-1)/(2T)]$ is simply the thermodynamic factor related to power. Knowing Z_1^+ and Z_1^- this system is numerically solvable and can give us an exact solution.

III. ANALYTICAL STUDY OF EQUALITY FOR THE RANDOM AND SCALE FREE GRAPHS

A. Random graph

The degree and conditional degree distributions for the Erdős-Rényi model are given by [3]

$$P_z = \binom{N-1}{z} p^z (1-p)^{N-1-z}, \tag{11}$$

$$P(z'|z) = P_{z'}, \tag{12}$$

which are well approximated by a Poisson distribution when $N \to \infty$ keeping Np constant. Then by (6) the distribution of wealth vs opportunity is given, at small T and large $\bar{w}$, by

$$\langle w_z \rangle = \frac{z}{2}\left[1 + \mathrm{erf}\left(\frac{z-\bar{z}}{\sqrt{2\bar{z}}}\right)\right], \tag{13}$$

which can be used to to find analytical expressions for the Gini index for this graph, of the order of 0.35 in the limit of large $\bar{w}$. Here erf(x) denotes the error function.

If one considers now the law of the jungle case with P_z given by Eq. (11) one can show that the critical coordination z_c is close to $\bar{w}$ and a numerical estimation of the Gini index in the limit of large average wealth returns a value of 0.51. Another interesting quantity is the proportion of the have nots in the society which also converges to 0.5 when $\bar{w}$ becomes large.

B. Scale free graph

1. Connected graph

The Barabàsi-Albert model of minimal coordination m gives the following degree and conditional degree distributions [4, 5]

$$P_z = \frac{2\bar{w}(\bar{w}+1)}{z(z+1)(z+2)} \tag{14}$$

$$P(z'|z) = \frac{\bar{w}(z+2)}{zz'(z'+1)} - \frac{\bar{w}}{zz'}\binom{2\bar{w}+2}{\bar{w}+1}\frac{\binom{z+z'-2\bar{w}}{z'-\bar{w}}}{\binom{z+z'+2}{z'}}. \tag{15}$$

Equation (14) shows that the minimal coordination corresponds to half the average coordination and thus $m = \bar{w}$. As we noted in the text, this model leads to a simple expression for the average wealth at $T \to 0$, or

$$\langle w_z \rangle = z - \bar{w}. \tag{16}$$

From that we can find the exact expressions for the cumulative population and wealth which define the Lorenz

FIG. 1: The Gini index and the proportion of the completely dispossessed in the population vs. the average wealth for the different cases we have studied.

curve ($y = y(x)$)

$$\sum_{z'=x\bar{w}}^{z} P_{z'} = x(z) = 1 - \frac{\bar{w}(\bar{w}+1)}{(z+1)(z+2)}, \quad (17)$$

$$\frac{1}{\bar{w}} \sum_{z'=\bar{w}}^{z} P_{z'} \langle w_{z'} \rangle = y(z) = 1 - \frac{\bar{w}+1}{z+2}\left(2 - \frac{\bar{w}}{z+1}\right). \quad (18)$$

Solving (17) and using (18) we get the Lorenz curve and the Gini index

$$y(x) = 1 - \frac{4(\bar{w}+1)}{1+\sqrt{1+\frac{4\bar{w}(\bar{w}+1)}{1-x}}}\left(1 - \frac{\bar{w}}{-1+\sqrt{1+\frac{4\bar{w}(\bar{w}+1)}{1-x}}}\right), \quad (19)$$

$$\text{Gini} = 1 - 2\int_0^1 y(x)dx \underset{\bar{w}>>1}{\rightarrow} \frac{2}{3}. \quad (20)$$

2. *Law of the jungle*

For disconnected agents of distribution of opportunities P_z given by Eq. (14) the critical coordination and average wealth are given by (for $T \to 0$)

$$z_c = 2\bar{w} + 1, \quad (21)$$

$$\langle w_z \rangle = z\,\mathcal{H}(z - 2\bar{w}), \quad (22)$$

where $\mathcal{H}$ is the Heaviside function defined such that it is null in $2m$. From that the Lorenz curve can be computed to be

$$\frac{1}{\bar{w}} \sum_{z'=\bar{w}}^{z} P_{z'} \langle w_{z'} \rangle = y'(z) = 1 - \frac{2(\bar{w}+1)}{(z+1)(z+2)}, \quad (23)$$

$$y'(x) = 1 - \frac{4(\bar{w}+1)}{1+\sqrt{1+\frac{4\bar{w}(\bar{w}+1)}{1-x}}}. \quad (24)$$

With these results one can show that the lower bound for the Gini index reached at large $\bar{w}$ is 0.83. The proportion of the dispossessed is simply the cumulative population evaluated in $2\bar{w}$, and from Eq. (14) it is given by $(3\bar{w} + 2)/(2(2\bar{w} + 1))$ which goes to 3/4 in the limit of large average wealth. We sum up the results obtained in this section in Fig 1.

IV. EFFECT OF FRUSTRATION-BASED INITIATIVE ON CO-EVOLUTION.

Consider three agents (a, b, c) and the transition $\{a - b, c\} \to \{a - c, b\}$. We model the competition of power and frustration in such a transition by choosing the probability p for it to occur as proportional to

$$p = \exp[\Delta\mathcal{P}/T + K((w_b - w_c)f_a + (w_c - w_a)f_c - w_a f_b)], \quad (25)$$

where $\Delta\mathcal{P}$ is the variation of power between the two configurations and the w_i's and f_i's ($i \in \{a, b, c\}$) are the wealth and frustration of the different agents. The presence of the power term assures that at the end of the move the wealth is assigned to maximize power.

To help understand this formula we report the following table which gathers extreme but informative examples

f_a	f_b	f_c	$p\exp[-\Delta\mathcal{P}/T]$	P/R/U
0	0	0	1	U
0	0	1	$\exp[-Kw_a]$	R
0	1	0	$\exp[-Kw_a]$	R
0	1	1	$\exp[-2Kw_a]$	R
1	0	0	$\exp[K(w_b - w_c)]$	P($w_b > w_c$), R($w_b < w_c$)
1	0	1	$\exp[K(w_b - w_a)]$	P($w_b > w_a$), R($w_a < w_c$)
1	1	0	$\exp[-Kw_c]$	R
1	1	1	1	U

where P, R and U stand for Promoted, Repressed and Unchanged. We see that most for the transitions are repressed, which reflects the social friction introduced by this formula. On the other hand two of them can be promoted: if a is very frustrated and if b is richer than c then a will be encouraged to associate with c, whatever its frustration, because he/she will have more chances to get the wealth from this operation. Of course if the three agents are equally frustrated then their frustration has no effect on the transition.

V. CAPTION OF ANIMATIONS

A. Animation 1

In this movie we illustrate the co-evolution of a graph for different initiative ($KT = 0.8$, 1, 1.6, 1.7) which corresponds to the four different observed regimes: Power dominated, power damped, cyclical and frustration dominated. We started the Monte Carlo simulations with a power-equilibrated random graph of 1000 nodes and average wealth $\bar{w} = 50$. The left panel corresponds to the temporal evolution of the average power, the Gini index and the average frustration in the system. From left to right the different columns show the shape of the degree distribution, the distribution of wealth vs. opportunity with error bars corresponding to the fluctuations due to the topological structure of the network and the Lorenz curve.

B. Animations 2 & 3

To assess the dependence of the co-evolution from initial condition we compare, in these two movies, the cases of $KT = 1.5$ starting from two different initial conditions. The first one is, as in the first movie, an Erdős Rényi graph with 1000 nodes and average wealth 50, the second is a non-equilibrated Bàrabasi-Albert graph with the same parameters. On top left we show the degree distribution of the networks, the top left corresponds to the wealth vs. opportunity distribution, bottom left is the evolution of power with time and bottom right is the Lorenz curve with the Gini index. We see that both these initial conditions lead to an oscillatory regime with in fact quite the same observable distribution. This and other simulations make us speculate that the dynamical evolution provided by our algorithm is not strongly dependent on the initial conditions.

[1] L. Pauling, Journal of the American Chemical Society **57**, 2680 (1935). I

[2] C. Nisoli, New Journal of Physics **16**, 113049 (2014), URL `http://stacks.iop.org/1367-2630/16/i=11/a=113049`.

[3] P. Erdős and A. Rényi, Acta Mathematica Hungarica **12**, 261 (1961).

[4] A.-L. Barabási and R. Albert, science **286**, 509 (1999).

[5] B. Fotouhi and M. G. Rabbat, The European Physical Journal B **86**, 1 (2013).
