# Supplementary figures and images for: Emergent inequality and self-organized social classes in a network of power and frustration
Source: PLoS One. 2017 Feb 17;12(2):e0171832. doi: 10.1371/journal.pone.0171832 (PMC5315399; doi:10.1371/journal.pone.0171832)

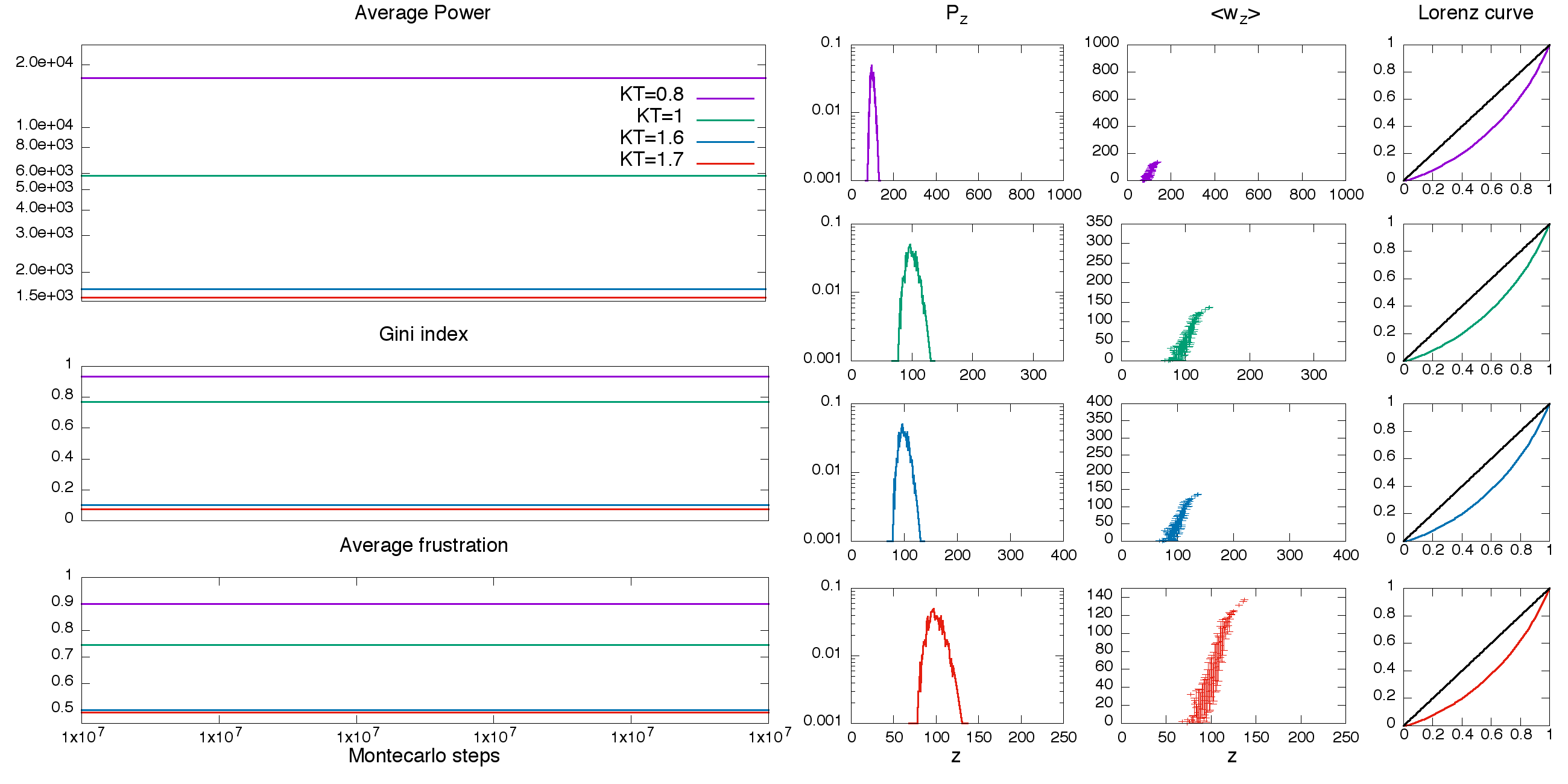

Supplement: S1 Animation — We started the Monte Carlo simulations with a power-equilibrated random graph of 1000 nodes and average wealth w¯=50. The left panel corresponds to the temporal evolution of the average power, the Gini index and the average frustration in the system. From left to right the different columns show the shape of the degree distribution, the distribution of wealth vs. opportunity with error bars corresponding to the fluctuations due to the topological structure of the network and the Lorenz curve. (GIF) [file pone.0171832.s002.gif]

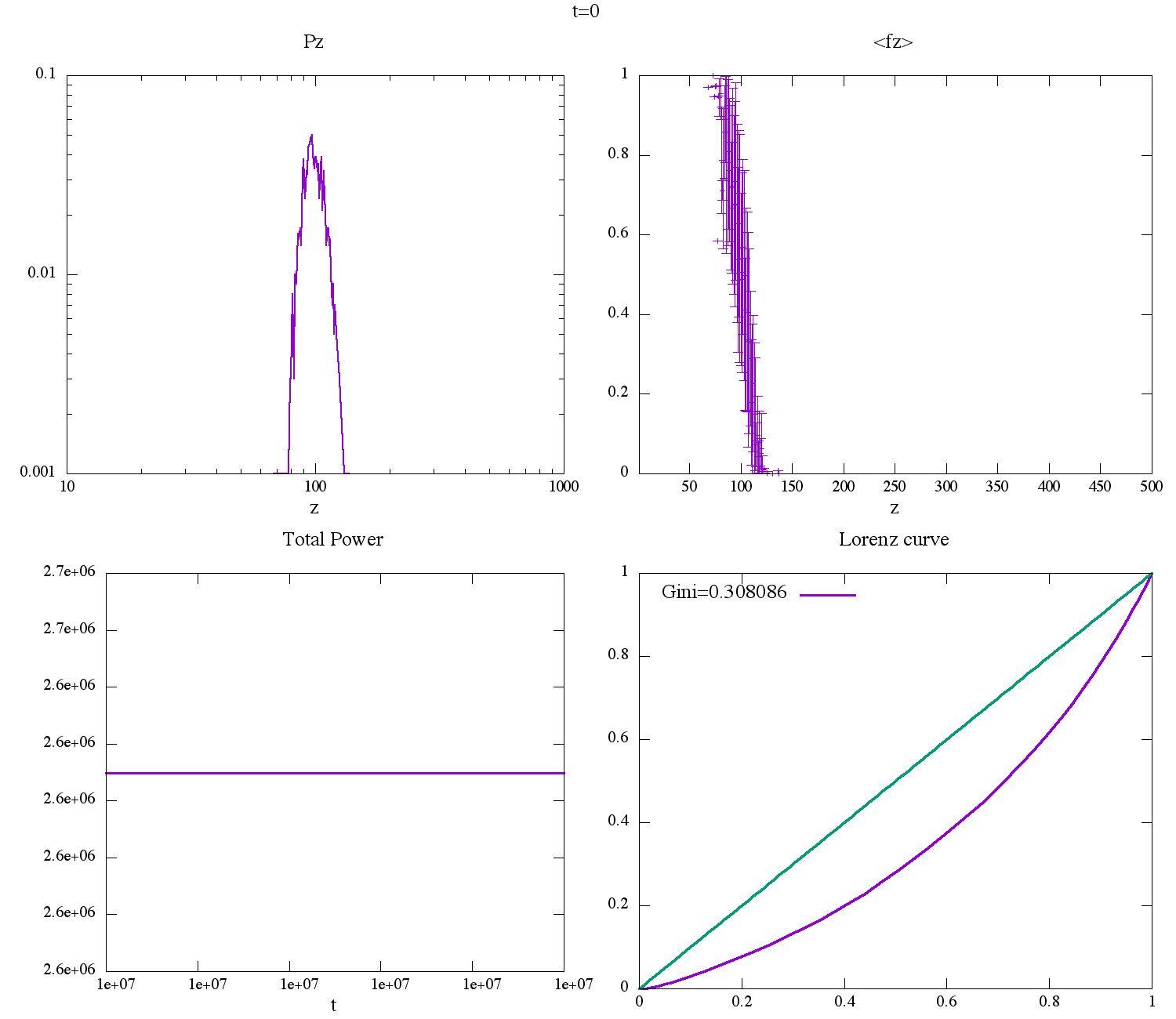

Supplement: S2 Animation — In this case we have an Erdős Rényi graph with 1000 nodes and average wealth 50, the second is a non-equilibrated Bàrabasi-Albert graph with the same parameters. On top left we show the degree distribution of the networks, the top left corresponds to the wealth vs. opportunity distribution, bottom left is the evolution of power with time and bottom right is the Lorenz curve with the Gini index. We see that both these initial conditions and those of S2 Animation lead to an oscillatory regime with in fact quite the same observable distribution. This and other simulations allow us to speculate that the dynamical evolution provided by our algorithm is not strongly dependent on the initial conditions. (GIF) [file pone.0171832.s003.gif]

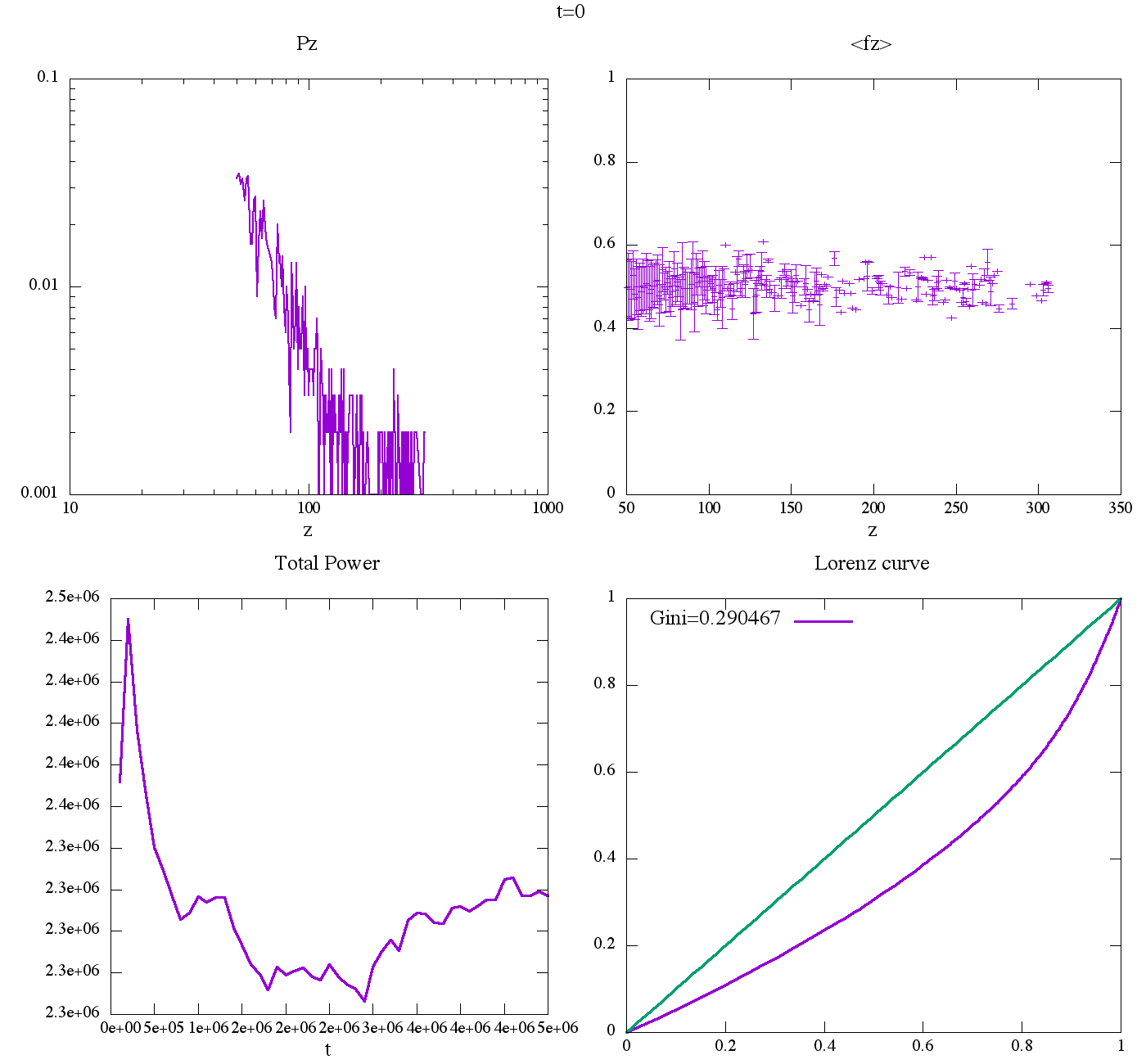

Supplement: S3 Animation — In this case we have a non-equilibrated Bàrabasi-Albert graph with the same parameters. On top left we show the degree distribution of the networks, the top left corresponds to the wealth vs. opportunity distribution, bottom left is the evolution of power with time and bottom right is the Lorenz curve with the Gini index. We see that these initial conditions lead to an oscillatory regime with in fact quite the same observable distribution. This and other simulations make us speculate that the dynamical evolution provided by our algorithm is not strongly dependent on the initial conditions. (GIF) [file pone.0171832.s004.gif]

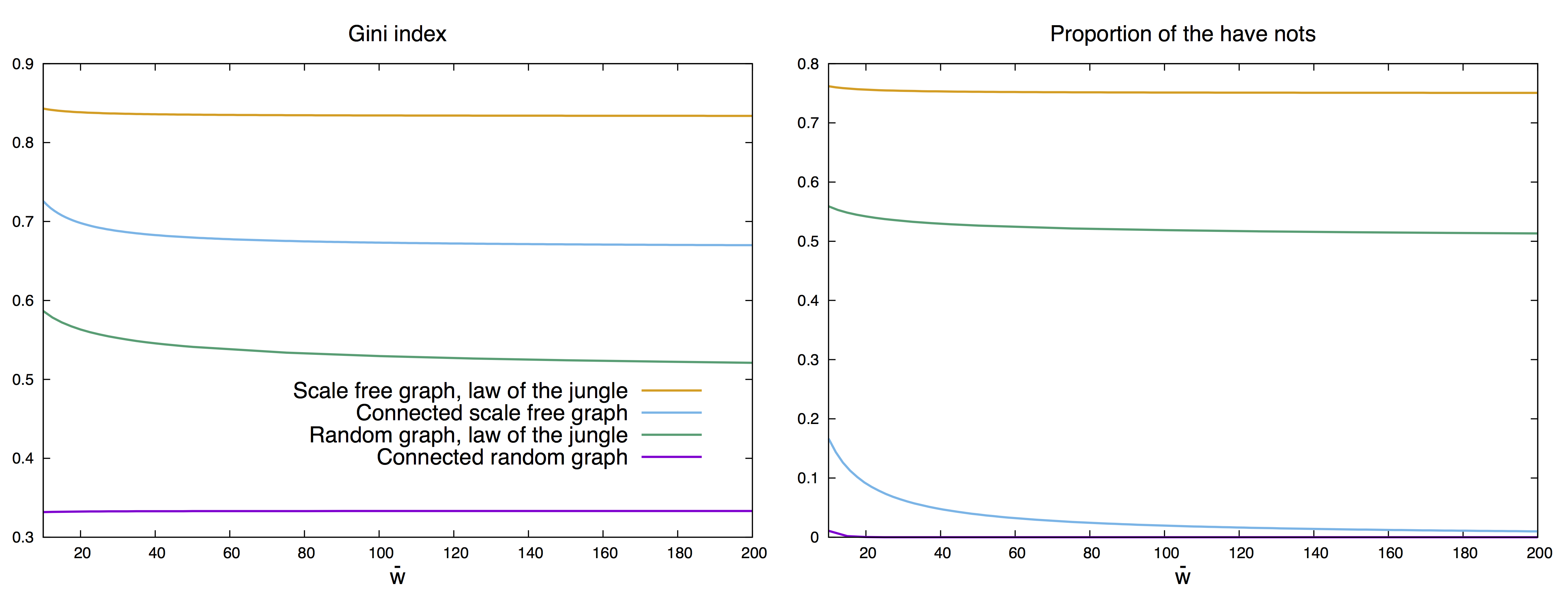

Supplement: S1 Fig — (TIFF) [file pone.0171832.s005.tiff]
